# Compliance of Clinical Trial Protocols for Foods with Function Claims (FFC) in Japan: Consistency between Clinical Trial Registrations and Published Reports

**DOI:** 10.3390/nu14010081

**Published:** 2021-12-25

**Authors:** Hiroharu Kamioka, Hideki Origasa, Jun Kitayuguchi, Kiichiro Tsutani

**Affiliations:** 1 Faculty of Regional Environment Science, Tokyo University of Agriculture, 1-1-1 Sakuragaoka, Setagaya-ku, Tokyo 156-8502, Japan; 2 Division of Biostatistics and Clinical Epidemiology, School of Medicine, University of Toyama, 2630 Sugiya, Toyama 930-0194, Japan; origasahideki@gmail.com; 3 Physical Education and Medicine Research Center Unnan, 328 Uji, Unnan City 699-1105, Japan; junk_907@yahoo.co.jp; 4 Facult of Health Sciences, Tokyo Ariake Medical and Health Sciences University, 2-9-1 Ariake, Kouto-ku, Tokyo 135-0063, Japan; tsutanik@gmail.com

**Keywords:** clinical trial registration, protocol, compliance, foods, randomized controlled trial

## Abstract

Background: A new type of foods with a health claims notification system, the Foods with Function Claims (FFC), was introduced in Japan in April 2015. This cross-sectional study sought to clarify compliance of clinical trial protocols reported as the scientific basis of efficacy in the FFC system. Methods: All articles based on clinical trials published on the Consumer Affairs Agency website from 1 July 2018 to 30 June 2021 were reviewed. Items assessed included first author characteristics (for-profit or academia), journal name, year published, journal impact factor in 2020, article language, name of clinical trial registration (CTR), and seven compliance items (Title: T, Participant: P, Intervention: I, Comparison: C, Outcome: O, Study design: S, and Institutional Review Board, IRB). Among studies that conducted CTR, consistency with these seven compliance items was evaluated. Results: Out of 136 studies that met all inclusion criteria, 103 (76%) performed CTR, and CTR was either not performed or not specified for 33 (24%). Compliance between the protocol and the text was high (≥96%) for items P and S, but considerably lower for items T, I, C, O, and IRB (52%, 15%, 13%, 69%, and 27%, respectively). Furthermore, 43% of protocols did not include functional ingredients or food names in items T or I. The total score was 3.7 ± 1.1 pts (out of 7). Conclusions: Some CTs had no protocol registration, and even registered protocols were suboptimal in transparency. In addition to selective reporting, a new problem identified was that the content of the intervention (test food) was intentionally concealed.

## 1. Introduction

The Codex Alimentarius Commission (CAC) is an intergovernmental organization that was founded in 1962 by the Food and Agriculture Organization (FAO) and the World Health Organization (WHO) [1]. The basic principles of CAC are that health claims should be substantiated by currently sound and sufficient scientific evidence, provide truthful and non-misleading information that consumers can use to choose healthy diets, and be supported by specific consumer education [2]. In accordance with CAC guidelines, only government-permitted Foods for Specified Health Uses (FOSHU) and foods with nutrient function claims (FNFC) can make function claims on food labels in Japan, and these must comply with specifications and standards designated by the government [3]. The FNFC can be used to supplement or complement the daily requirement of nutrients (vitamins, minerals, etc.) that tend to be insufficient in an everyday diet. The FOSHU are scientifically accepted for their usefulness in maintaining and promoting health and are therefore permitted to contain food effects and safety claims that have been evaluated by the government.

**Figure 1:**
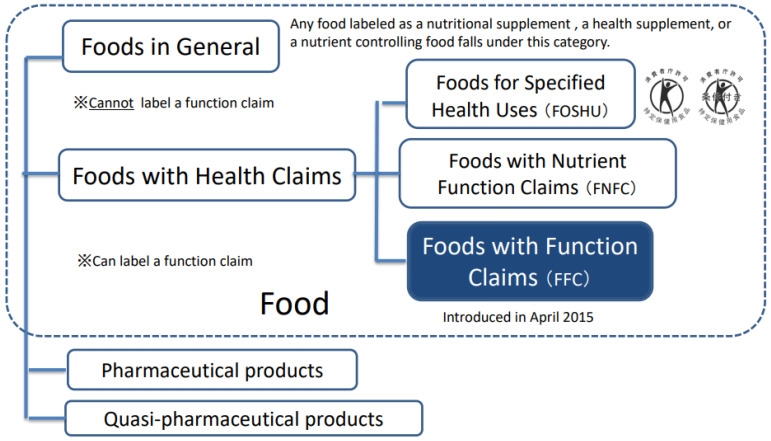
Food labeled with certain nutritional or health functions in Japan (modified partially for this study based on the Consumer Affairs Agency website in Japan). The new system (FFC) allows labeling, which indicates that the food is expected to have a specific effect on health, except for reducing the risk of diseases, through the process of submission to the Secretary-General of the Consumer Affairs Agency in Japan.

In addition to these categories, a new type of foods with health claims, called Foods with Function Claims (FFC), was introduced in April 2015 (Figure 1). The FFC allows manufacturers to submit labeling to the Secretary-General of the Consumer Affairs Agency (CAA) in Japan that indicates the food is expected to have a specific effect on health. Unlike the strict evaluation criteria applied through the FOSHU and FNFC processes, the FFC is only a notification system in which food manufacturers must meet five specific criteria. Although the government does not evaluate the safety and effectiveness of the submitted product, i.e., it does not utilize a notification system, the industry (applicant) must fulfill several procedures to submit a notification. All the FFC criteria submitted by the manufacturers are disclosed on the CCA website, which gives approval for the labeling of food products. For a food product to claim effectiveness on its label, evidence for its proposed function claims must be substantiated by one of two standard scientific methods: clinical trials (CTs) such as randomized controlled trials (RCTs) or systematic reviews (SRs). Detailed guidelines about the use of these two methods for the FFC have been published on the CAA website [4].

The problem with the FFC notification system, which is the adequacy of research reports, has been highlighted by the CAA and by research groups. The CAA examined 50 reported CTs and determined that many had inappropriate protocols and methods for evaluating the risk of bias and also had conflicts of interest [5]. Tanemura et al. identified problems with the reporting quality and associated issues for RCTs of the FFC [6]. There was insufficient information on items associated with sample size, allocation and blinding, results of outcomes and estimation, generalizability of the results, and study registration numbers. To protect consumers, these reports suggested that researchers should monitor and confirm that referenced RCTs are above a certain level of quality.

Helsinki Declaration (2013) declared that “Every research study involving human subjects must be registered in a publicly accessible database before recruitment of the first subject” [7]. Clinical trial registration (CTR) of CTs is extremely important for promoting transparency and integrity in medical research and helps to ensure a complete and non-biased record of all CTs [8,9,10,11]. Recommendations for the Conduct, Reporting, Editing, and Publication of Scholarly Work in Medical Journals by the International Committee of Medical Journal Editors (ICMJE) require CTR of CTs in all medical journals [12]. The purpose of CTR is to (i) prevent selective publication and selective reporting of research outcomes, (ii) prevent unnecessary duplication of research effort, (iii) help patients and the public know what trials are planned or ongoing into which they might want to enroll, and (iv) help give ethics review boards considering approval of new studies a view of similar work and data relevant to the research they are considering. Especially, selective reporting of research outcomes (e.g., replacing secondary outcome with the primary outcome or not defining primary outcome) could seriously mislead readers [8,10,13,14,15]. Considering the limitations of the FFC notification system, we focused on compliance of CT protocols, i.e., consistency between registered and reported material in a CT. 

The purpose of this cross-sectional study was to clarify the compliance of CT protocols reported as the scientific basis of efficacy in the FFC system. Based on results, questions about the following issues necessary to improve CT protocol compliance were clarified: (i) What percentage of studies have pre-registered clinical trials? How many protocols are available for unregistered studies? (ii) What is the degree of compliance, i.e., the degree of consistency about PICOS* between the protocol and the description of articles? What are the items and their characteristics that often violate the protocol? (iii) Is compliance correlated with author characteristics (industry authors, academia authors), the year in which the paper was published, the relationship between English and other languages, and the journal impact factor? 

## 2. Materials and Methods

### 2.1. Eligibility and Exclusion Criteria (Target Article)

**Figure 2:**
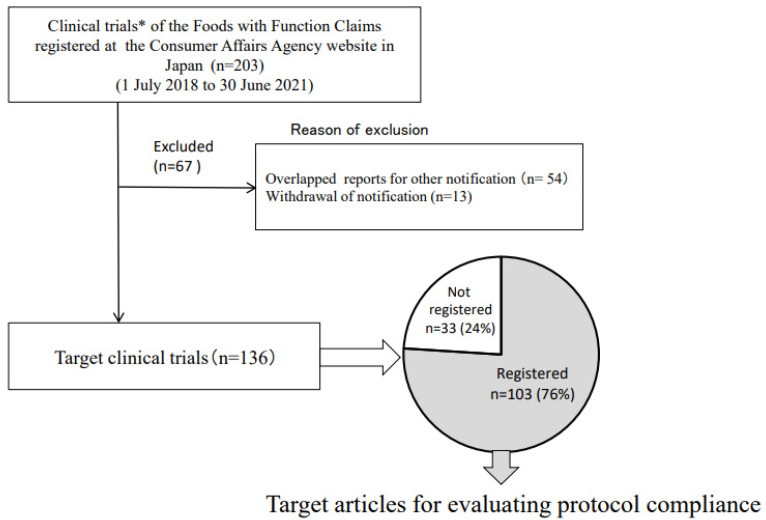
Flow of trial process and implementation status of clinical trial registration. * Number of notifications as Foods with Function Claims (*n* = 177).

All reported articles based on CTs published on the CAA website during the three years from 1 July 2018 to 30 June 2021 were reviewed. Articles about systematic reviews and observational studies were excluded from the review. When there were duplicated articles, such as multiple notifications using the same article, only the first article was adopted. There were 177 applicable notifications and 203 articles that met the first eligibility criteria (Figure 2, Supplementary Table S1).

### 2.2. Data Extraction Source and Data Items

We downloaded target articles from the CAA website and also obtained protocols from CTR described in each article. If an article noted that registration was not underway but the protocol existed, we specified that information.

The following data items were collected: first author characteristics (company-based/profit-based researchers, academia researchers, or other), journal name, year published, journal impact factor (IF) in 2020, language of the article, name of CTR, and seven compliance items (Title: T, Participant: P, Intervention: I, Comparison: C, Outcome: O, and Study design: S, and Institutional Review Board, IRB). We assessed the IF according to the Clarivate Analytics’s gate (https://jcr.clarivate.com/ (accessed on 10 November 2021)).

One point was given if it was implemented/described, and zero points if there was no description or there was a discrepancy between the protocol and the content described in an article. A total of seven points were given, which was used as the quality score.

### 2.3. Measures and Statistical Analysis

Among the studies that conducted CTR, consistency with the above-mentioned seven items was assessed. In addition, statistical association of each study was analyzed using the quality score as the dependent variable and author characteristics, year of publication (before 2017 or after 2018), language characteristics (Japanese or English), and IF as explanatory variables. Since the IF and quality score were not normally distributed, intergroup comparisons were performed with the nonparametric Mann–Whitney test and shown in a boxplot. However, in the results presented in the text, the numerical values for comparison between groups are shown by mean ± standard deviation for easy understanding. For the relationship between IF and compliance score, Spearman’s rank correlation coefficient was used.

IBM SPSS Statistics 20.0 (IBM Corporation, Armonk, NY, USA) was used for statistical analyses. A *p*-value less than 0.05 was considered statistically significant.

### 2.4. Protocol Registration

The study methodology (protocol) was established on 4 October 2021 and partially revised on 2 November 2021. The study was registered as UMIN 000045685 by the University Hospital Medical Information Network Clinical Trials Registry (UMIN-CTR)* in Japan (refer: https://upload.umin.ac.jp/cgi-open-bin/ctr/ctr_view.cgi?recptno=R000052143). However, UMIN-CTR could not register the contents of all the protocols in the input settings, so the complete protocol was stored in an online cloud. It can be viewed from this link: https://drive.google.com/file/d/1A_p1OokEVXpisvEsoQkrkWfUtEpGl2Pq/view?usp=sharing.

* UMIN-CTR is the largest CTR in Japan and joined the WHO registry network in October 2008. 

## 3. Results

A total of 136 studies met all inclusion criteria (Figure 2, Supplementary Table S2). Of these, 103 (76%) performed CTR, and 33 (24%) did not perform CTR or did not specify whether or not CTR was performed. Table 1 (and Supplementary Table S1) show that UMIN-CTR was the most used CTR in 101 (98%) of the articles. In matchings between protocols and the articles’ text, compliance items P and S were high at 96% or greater, but items T, I, C, O, and IRB were considerably lower (52%, 15%, 13%, 69%, and 27%, respectively). Furthermore, 43% of the protocols did not include information on functional ingredients or food names in items T or I. The quality score was 3.7 ± 1.1 pts (out of 7), and there was only one paper with a total score of 7 points.

Eligible articles were published in 27 journals, and most (62%) were published in 2018–2019 (Table 2). The languages of eligible publications were English (55%) and Japanese (45%). According to the affiliation classification of the first author, for-profit was 81% and academia was 19%. Seventy-four percent of journals had no IF. 

**Figure 3:**
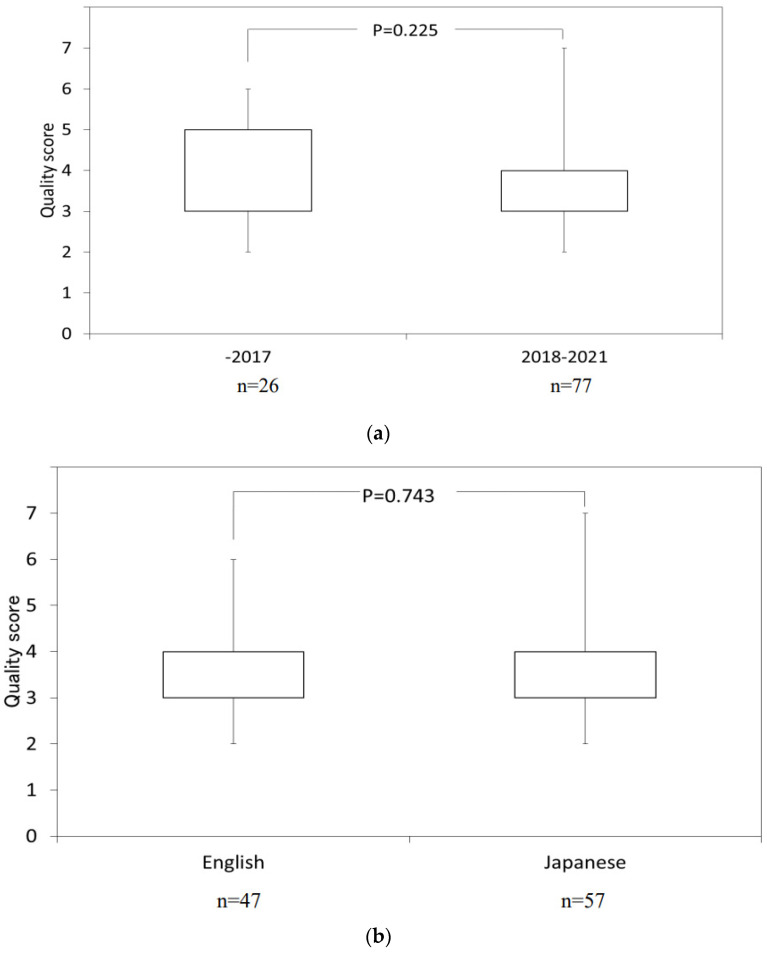

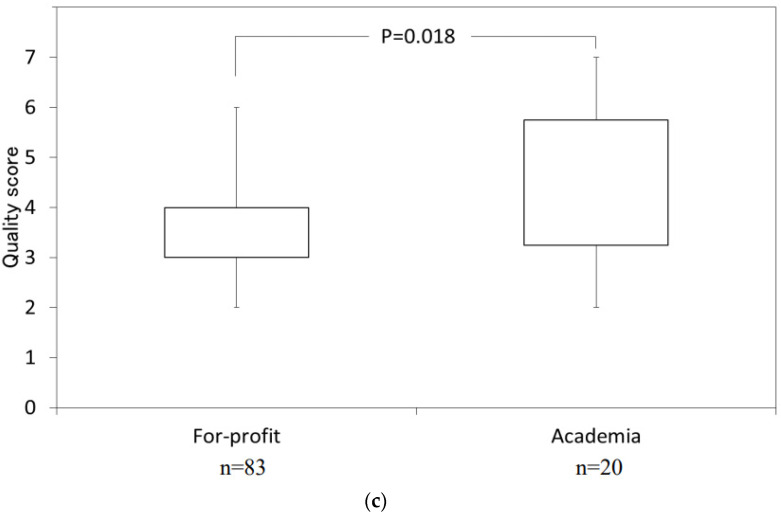
(**a**) Quality score during the period of published year. Quality score was defined as a sum of the seven dichotomous items. Boxplot. Mann–Whitney test. *p*-value less than 0.05 was considered statistically significant. (**b**) Quality score between Japanese and English publications. Quality score was defined as a sum of the seven dichotomous items. Boxplot. Mann–Whitney test. *p*-value less than 0.05 was considered statistically significant. (**c**) Quality score for category of first author’s organization. Quality score was defined as a sum of the seven dichotomous items. Boxplot. Mann–Whitney test. *p*-value less than 0.05 was considered statistically significant.

**Figure 4:**
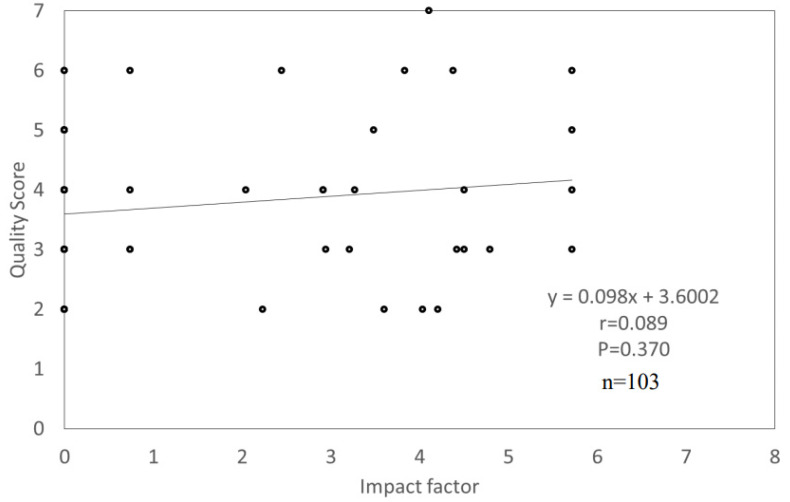
Correlation between journal’s impact factor and quality score. Spearman’s rank correlation coefficient. *p*-value less than 0.05 was considered statistically significant.

There was no significant difference (*p* = 0.225) in scores between the published year categories of 2015–2017 (4.0 ± 1.4 pts) and 2008–2021 (3.8 ± 1.2 pts) (Figure 3a). There was also no significant difference (*p* = 0.743) in scores between English (3.8 ± 1.2 pts) and Japanese (3.6 ± 1.0 pts) language publications (Figure 3b). However, scores were significantly different (*p* = 0.018) depending on whether the first author’s organization was for-profit (3.5 ± 1.0 pts) or academia (4.0 ± 1.3 pts) (Figure 3c). A significant correlation (*p* = 0.089) between IF and score was not found with Spearman’s rank correlation coefficient; r = 0.370 (Figure 4).

## 4. Discussion

This was the first study to clarify the compliance of CT protocols of the FFC in Japan; unfortunately, it became clear that there were still some reports in which the protocol was not registered, or its registration status was not mentioned in the paper. Even if CTR was implemented, many protocols were suboptimal in transparency. In addition to the selective reporting of CTs that has been previously identified, we found that a new research methodology problem in this field was that nearly half of CTs concealed the intervention content (test food).

### 4.1. Registration of Protocol

Despite the widespread endorsement of CTR globally, CTR in FFC remains suboptimal, with no registration or, in many cases, unknown existence of the protocol itself (24%). Our findings are consistent with previous studies that reported protocols were not fully registered [16,17,18,19,20,21]. Gopal et al. [18] concluded that unregistered trials were more likely to report favorable findings than were registered trials (89% vs. 64%, relative risk = 1.38, 95% confidence interval = 1.20–1.58; *p* = 0.004). No registration or absence of the protocol immediately creates doubt about the internal validity of results in a CT. Therefore, the validity of target articles in this study is unknown, especially if there were protocol violations [22], which could lead to flawed conclusions based on systematic errors and/or protocol deviations [23].

There are also few registrations in the fields of surgery [19] and addiction [20] or for trials in a single country’s health research [21], which could be because the journal’s regulation did not require registration as a prerequisite for submitting papers. The same might be inferred in the field of nutrition, so journals other than ICMJE member journals are also considered to have an urgent need to register protocols. The CTR was specified in the CONSORT 2010 statement [24,25] and the CONSORT 2010 statement: crossover extension [26], which describe the method for RCT reporting, and in SPIRIT 2013 [27], which summarizes the reports of CT protocols. In spite of the existence of these early guidelines, the oldest of the target articles in our study was published in 2015, and there were many relatively newer reports, which suggests that the importance of registration is not yet well understood by journals and researchers.

The FFC guideline stated that “research started within one year after the enforcement of the FFC system may be reported in a format that does not comply with international guidelines”. This special measure may have led to a disregard for the importance of the protocol [4]. In the case of industry funding, there is evidence that favorable results are more likely to be reported and that unfavorable results are difficult to report [28]. Almost all FFC notifications were from industries, and 80% of the authors of the target papers were affiliated with those industries, so this problem is worrisome.

### 4.2. Inconsistency with Protocol

There were discrepancies between registered and published primary outcomes (about 30%), which indicated there might be selective outcome reporting that has been previously described [8,10,13,14,15]. Neither compliance items T nor I contained detailed information on the test food in a quarter of the protocols. We could not even identify what the study was. This concealment must have been to prevent other competitors from stealing the intended food development content. In the author category, academia is not directly related to business, and this may be supported by the fact that academia had a significantly higher quality score than non-profit. In corporations, there are usually “trade secrets”, especially for new products. When researchers employed by the company conduct a CT, they may conceal the registration of contents because they recognize that the functional component of the product is confidential. In such cases, it is meaningless that CTR exists because it is unclear what kind of research was conducted. To remedy this situation, researchers need to re-familiarize themselves with the four dimensions of the purpose of the ICMJE registration policy [12]. 

The IRB omissions were present in as many as three-quarters of the articles. Many papers use UMIN-CTR, and the IRB column in this template is required as an item. All IRB information is included in the paper, so the reason for this mis-statement is unknown. On the other hand, there were good descriptions of items that would be convenient for competitors to browse, namely P and S.

### 4.3. Impact on SRs

For a food product to claim effectiveness on the FFC’s label, evidence for its proposed function claims must be substantiated by another standard scientific method, specifically SRs. In fact, under the FFC system, about 90% of notifications are from SRs [4]. Previous studies [29,30] that evaluated the quality of methodologies and reporting in SRs based on the FFC reported that there were very poor descriptions and/or implementation of study selection, data extraction, search strategy, evaluation methodology for risk of bias, assessment of publication bias, and formulating conclusions based on methodological rigor and scientific quality of the included studies. 

Furthermore, when SRs contain RCTs with inappropriate and biased protocols, such as those included in this study, the reliability of the FFC’s claim may be questionable. While SRs exceeding the number of RCTs have been published globally [31], high-quality CTs are required, and registration of the protocol is most essential.

### 4.4. Future Research Challenges to Improve the Compliance of Protocols on the FFC 

Table 3 summarizes the future challenges for CTs on the health enhancement effects of the FFC and related healthy foods. Regarding the food industry and for-profit researchers, they should reconfirm Helsinki Declaration and ICMJE policy and study the current standard guidelines for RCTs (i.e., CONSORT 2010 statement [24,25], CONSORT 2010 statement-crossover extension [26], and SPIRIT 2013 [27]) before research is conducted. In fact, in Japan, all of these guidelines have been translated into Japanese [32,33,34] and published for use by researchers who are unfamiliar with English. Academia researchers at Japanese universities and national research institutes regularly take research ethics education programs to prevent research misconduct. Such education, while not essential for industry, transcends economic objectives and may be necessary to improve protocol compliance.

For regulators, even if they have notification systems, they should evaluate not only the formal confirmation in the article but also any differences from or deficiencies with the protocol. If they identify any problems, they should seek clarification from the submitter.

In regard to the journal’s editors and peer-reviewers, they should assess the consistency between registration and article contents before making decisions about publication. Prior studies indicate that these editors and reviewers often do not take advantage of the possibilities provided by registration [16,35,36,37]. For example, van Lent et al. reported that differences between trial information specified in registries and that reported in submitted manuscripts were not a decisive factor for manuscript rejection after initial editorial screening or after peer review [37]. As mentioned above, in food research, in addition to selective reporting, it is essential to check the content of interventions described in the protocol.

As a new tool for promoting registration and its effective operation, a blockchain solution using smart contracts has been proposed for managing data and process workflow in CTs [38]. This would be a working proof-of-concept solution to address the data management, protocol compliance, transparency, and data integrity challenges in a CT. In the future, CTR may move to such a comprehensive method.

### 4.5. Limitations

There were several limitations to the present study. First, we evaluated only some items in the protocol and could not follow all items of the ICMJE registration policy. For example, the guidelines require CTs to be publicly and prospectively registered to be considered for publication within an ICMJE member journal, i.e., investigators need to register their trials in one of the public trial registries before enrolment of the first participant [7,12]. A recent study [39] clarified that CTs published in ICMJE member journals were highly compliant, but prospective trial registrations were still low. Furthermore, with regard to CTR after completion of a study, recent studies found that faster registration has become widespread [40,41]. These two factors are important for showing transparency in registration requirements. However, because we did not set our protocol to assess for these factors, we could not clarify their impact on our results. 

Second, we focused only on CTs based on notification to the FFC in Japan (a single country), so our finding may not necessarily be generalizable to all CTs of healthy foods. In fact, about half of the articles in our study were written in Japanese. Third, the 133 articles included in our study were a relatively medium sample size and limited to three years of CTs, so we cannot be certain how representative they were of all FFC notifications to the CAA. Fourth, a common weakness of our cross-sectional study design is that there can be differences in quality between CTs conducted before and after the timeframe of this study because of time lag. Our study provides only an early look at the effects of trial registration on results and conclusions in a particular sample of registered and unregistered trial reports. In Japan, compliance with research ethics guidelines, CONSORT 2010 and SPIRIT 2013 has been greatly improving year by year, and new CTs after 2021 may have improved protocol quality.

Fifth, since we didn’t use the IF of published year for each article, the use of the IF for the year 2020 is somewhat difficult to understand about the relationship with the year of publication of the article. Sixth, this study did not evaluate the risk of bias. Statistical considerations for bias and protocol deviation have been previously evaluated [42], so this becomes an issue to be addressed for the next stage of our study. Seventh, we could not fully describe the complete list of products identified in our study (i.e., identified by product name and food business operator’s name) because of the potential risk of civil suits and other legal troubles. 

Finally, in our research protocol, the title listed in the CTR was added as a new item after the protocol was fixed. When we started checking each CTR, we noticed that there was either unclear or no mention of the test food in compliance item I or in the title. This suggests that there is a potentially significant problem if the viewer cannot understand what kind of CT was conducted. 

## 5. Conclusions

There were CTs for which the protocol had not been registered in the FFC, and even the registered protocols were suboptimal in transparency. In particular, in addition to selective reporting, a new problem identified was that the content of the intervention (test food) was intentionally concealed.

It is necessary to raise awareness that protocols for food-related CTs need to be described carefully without masking specific information so that details of the research can be recognized. Journal editors and peer reviewers in this field should scrutinize differences between and ambiguities of the submitted manuscript and its protocol. In addition, regulators should confirm the protocol before accepting it, even if it is included in a notification system.

## Figures and Tables

**Table 1:** Utilized clinical trial registration and protocol compliance.

Name of Clinical Trial Registration	
UMIN-CTR	101 (98%)
ANZCTR	1 (1%)
ClinicalTrials.gov	1 (1%)
Protocol item	
(Number of articles with a good quality with percentage)	
Title	54 (52%) **
Participant	99 (96%)
Intervention	16 (15%) **
Comparison	14 (13%)
Outcome	71 (69%)
Study design	101 (98%)
Institutional Review Board	28 (27%)
Quality score (pts) *	3.7 ± 1.1 [2,3,4,5,6,7]

Value: n (%). * mean ± standard deviation [range]. ** No. (%) of papers with insufficient description for both title and intervention: 44 (43%).

**Table 2:** Published journal’s characteristics.

Journal Name	
薬理と治療/Japanese Pharmacological and Therapeutics	57 (55%)
診療と新薬/Medical Consultation and New Remedies	9 (9%)
応用薬理/Pharmacometrics	4 (4%)
Functional Foods in Health and Disease	4 (4%)
Nutrients	4 (4%)
Diabetes, Metabolic Syndrome and Obesity: Targets and Therapy	2 (2%)
Frontiers in Neuroscience	2 (2%)
Integrative Molecular Medicine	2 (2%)
American Journal of Geriatric Psychiatry	Common to all of the following journals: 1 (1%)
Applied and Environmental Microbiology	
機能性食品と薬理栄養/Associate Journal of Japanese Society for Medical Use of Functional Foods	
Benefical Microbes	
Biological and Pharmaceutical Bulletin	
Bioscience, Biotechnology, and Biochemistry	
Bioscience of Microbiota, Food and Health	
Complementary Therapies in Medicine	
Glycative Stress Research	
International Journal of Food Sciences and Nutrition	
Journal of Clinical Biochemistry and Nutrition	
Journal of Dairy Science	
日本栄養･食糧学会誌/Journal of Japanese Society of Nutrition and Food Science	
Journal of Traditional and Complementary Medicine	
Neurogastroenterology and Motility	
Nutrition Journal	
調理食品と技術/Prepared Foods and Technology	
Science Reports	
Skin Pharmacology and Physiology	
Published year	
2014–2015	1 (1%)
2016–2017	25 (24%)
2018–2019	64 (62%)
2020–2021	13 (13%)
Language	
English	56 (55%)
Japanese	47 (45%)
Category of first author’s organization	
For-profit	83 (81%)
Academia	20 (19%)
Journal’s impact factor in 2020	
None (0)	76 (74%)
1.999>	4 (4%)
2.000–3.999	11 (11%)
>4.000	12 (12%)

Value: n (%).

**Table 3:** Future challenges to improve protocol compliance in the food-related clinical trials.

For food industry and researcher	
#1	Researchers should be based on Helsinki Declaration and ICMJE policy.
#2	Researchers should be based on some reporting guidelines: CONSORT 2010 and its extension, and SPIRIT 2013.
#3	They should receive regular research ethics education, as do academia researchers at universities and national research institutes.
For regulator (e.g., Consumer Affairs Agency in Japan)	
#4	Even if notification system is based on submitter’s own responsibility, it is necessary to confirm consistency with the protocol and obtain responses to any findings about differences or deficiencies.
For journal’s editor and peer-reviewer	
#5	They should scrutinize the information based on registration and make a decision on publication.

## Data Availability

Not applicable.

## Supplementary Materials

The following are available online at https://www.mdpi.com/article/10.3390/nu14010081/s1, Table S1: Characteristics of clinical trials of the Food with Function Claims Table S2: Category of first author, language, and protocol compliance.

## Abbreviations

CAA	Consumer Affairs Agency
CAC	Codex Alimentarius Commission
CT	Clinical Trial
CTR	Clinical Trial Registration
FAO	Food and Agriculture Organization
FFC	Foods with Function Claims
FNFC	Foods with Nutrient Function Claims
FOSHU	Foods for Specified Health Uses
ICMJE	International Committee of Medical Journal Editors
IF	Impact Factor
IRB	Institutional Review Board
PICOS	Participant, Intervention, Comparison, Outcome, and Study design
RCT	Randomized Controlled Trial
SR	Systematic Review
UMIN-CTR	University Hospital Medical Information Network Clinical Trials Registry
WHO	World Health Organization

## References

[1] The CODEX Alimentarius Committee (2021). About CODEX Alimentarius. http://www.fao.org/fao-who-codexalimentarius/about-codex/en/.

[2] The Ministry of Health, Labour, Welfare in Japan. http://www.mhlw.go.jp/english/topics/foodsafety/dna/02-01.html.

[3] The CODEX Alimentarius Committee Guidelines for Use of Nutrition and Health Claims. http://www.fao.org/fao-whocodexalimentarius/standards/list-of-standards/en/?provide=standards&orderField=fullReference&sort=asc&num1=CAC/GL.

[4] Consumer Affairs Agency, Government of Japan Guideline (Updated March 2021). https://www.caa.go.jp/policies/policy/food_labeling/foods_with_function_claims/assets/foods_with_function_claims_210322_0002.pdf.

[5] Consumer Affairs Agency, Government of Japan Verification of Scientific Evidence on “Foods with Function Claims”: Assessment of the Submitted Clinical Trials. https://www.caa.go.jp/policies/policy/food_labeling/foods_with_function_claims/pdf/foods_index_23_171025_0001.pdf.

[6] Tanemura N., Hamadate N., Urushibara H. (2018). Evaluation of randomized controlled trials of foods with functional claims request: The learning outcomes from studies in Japan. J. Funct. Foods.

[7] World Medical Association WMA Declaration of Helsinki-ethical Principles for Medical Research Involving Human Subjects; Research Registration and Publication and Dissemination of Results. https://www.wma.net/policies-post/wma-declaration-of-helsinki-ethical-principles-for-medical-research-involving-human-subjects/.

[8] Chan A.W., Hrobjartsson A., Haahr M.T., Gotzsche P.C., Altman D.G. (2004). Empirical evidence for selective reporting of outcomes in randomized trials: Comparison of protocols to published articles. JAMA.

[9] Chen R., Desai N.R., Ross J.S., Zhang W., Chau K.H., Wayda B., Murugiah K., Lu D.Y., Mittal A., Krumholz H.M. (2016). Publication and reporting of clinical trial results: Cross sectional analysis across academic medical centers. BMJ.

[10] Turner E.H., Matthews A.M., Linardatos E., Tell R.A., Rosenthal R. (2008). Selective publication of antidepressant trials and its influence on apparent efficacy. N. Engl. J. Med..

[11] Hopewell S., Loudon K., Clarke M.J., Oxman A.D., Dickersin K. (2009). Publication bias in clinical trials due to statistical significance or direction of trial results. Cochrane Database Syst. Rev..

[12] International Committee of Medical Journal Editors Recommendations for the Conduct, Reporting, Editing, and Publication of Scholarly Work in Medical Journals (Updated December 2019). icmje-recommendations.pdf.

[13] Kirkham J.J., Dwan K.M., Altman D.G., Gamble C., Dodd S., Smyth R., Williamson P.R. (2010). The impact of outcome reporting bias in randomized controlled trials on a cohort of systematic reviews. BMJ.

[14] Macleod M.R., Michie S., Roberts I., Dirnagl U., Chalmers I., Loannidis J.P.A., Salman R.A.S., Chan A.W., Glasziou P. (2014). Biomedical research: Increasing value, reducing waste. Lancet.

[15] Dal-Ré R., Ross J.S., Marusic A. (2016). Compliance with prospective trial registration guidance remained low in high-impact journals and has implications for primary end point reporting. J. Clin. Epidemiol..

[16] Mathieu S., Boutron I., Moher D., Altman D.G., Ravaud P. (2009). Comparison of registered and published primary outcomes in randomized controlled trials. JAMA.

[17] Dwan K., Altman D.G., Cresswell L., Blundell M., Gamble C.L., Williamson P.R. (2011). Comparison of protocols and registry entries to published reports for randomised controlled trials. Cochrane Database Syst. Rev..

[18] Gopal A.D., Wallach J.D., Aminawung J.A., Gonsalves G., Dal-Ré R., Miller J.E., Ross J.S. (2018). Adherence to the International Committee of Medical Journal Editors’ (ICMJE) prospective registration policy and implications for outcome integrity: A cross-sectional analysis of trials published in high-impact specialty society journals. BMC Med. Res. Methodol..

[19] Zhou J., Li J., Zhang J., Geng B., Chen Y., Zhou X. (2021). Requirements for study registration and adherence to reporting guidelines in surgery journals: A cross-sectional study. World J. Surg..

[20] Cooper C.M., Gray H., Barcenas L., Torgerson T., Checketts J.X., Vassar M. (2020). An evaluation of reporting guidelines and clinical trial registry requirements among addiction medicine journals. J. Am. Osteopath. Med..

[21] Clyne B., Boland F., Murphy N., Murphy E., Moriarty F., Barry A., Wallace E., Devine T., Smith S.M., Devane D. (2020). Quality, scope and reporting standards of randomised controlled trials in Irish Health Research: An observational study. Trials.

[22] Sweetman E.A., Doig G. (2011). Failure to report protocol violations in clinical trials: A threat to internal validity?. Trials.

[23] Ghooi R.B., Bhosale N., Wadhwani R., Divate P., Divate U. (2016). Assessment and classification of protocol deviations. Perspect. Clin. Res..

[24] Schulz K.F., Altman D.G., Moher D., CONSORT Group (2010). CONSORT 2010 statement: Updated guidelines for reporting parallel group randomised trials. PLoS Med..

[25] Moher D., Hopewell S., Schulz K.F., Montori V., Gøtzsche P.C., Devereaux P.J., Elbourne D., Egger M., Altman D.G. (2010). CONSORT 2010 explanation and elaboration: Updated guidelines for reporting parallel group randomised trials. BMJ.

[26] Dwan K., Li T., Altman D.G., Elbourne D. (2019). CONSORT 2010 statement: Extension to randomized crossover trials. BMJ.

[27] Chan A.W., Tetzlaff J.M., Altman D.G., Laupacis A., Gøtzsche P.C., Krleža-Jerić K., Hróbjartsson A., Mann H., Dickersin K., Berlin J.A. (2013). SPIRIT 2013 Statement: Defining standard protocol items for clinical trials. Ann. Intern. Med..

[28] Melander H., Ahlqvist-Rastad J., Meije G., Beermann B. (2003). Evidence b(i)ased medicine: Selective reporting from studies sponsored by pharmaceutical industry; review of studies in new drug applications. BMJ.

[29] Kamioka H., Tsutani K., Origasa H., Yoshizaki T., Kitayuguchi J., Shimada M., Tang W., Takano-Ohmuro H. (2017). Quality of systematic reviews of the foods with function claims registered at the Consumer Affairs Agency website in Japan: A prospective systematic review. Nutr. Res..

[30] Kamioka H., Tsutani K., Origasa H., Yoshizaki T., Kitayuguchi J., Shimada M., Wada Y., Takano-Ohmuro H. (2019). Quality of systematic reviews of the Foods with Function Claims in Japan: Comparative before- and after-evaluation of verification reports by the Consumer Affairs Agency. Nutrients.

[31] Niforatos J.D., Weaver M., Johansen M.E. (2019). Assessment of publication trends of systematic reviews and randomized clinical trials, 1995 to 2017. JAMA Intern. Med..

[32] 津谷喜一郎, 元雄良治, 中山健夫(訳) (2010). CONSORT 2010声明: ランダム化並行群間比較試験報告のための最新版ガイドライン. 薬理と治療.

[33] 上岡洋晴, 津谷喜一郎, 折笠秀樹 (2020). ｢CONSORT クロスオーバーExtension｣の紹介と解説. 薬理と治療.

[34] 折笠秀樹, 津谷喜一郎, 上岡洋晴(訳) (2017). SPIRIT 2013声明: 臨床試験のための標準的なプロトコール項目の規定. 薬理と治療.

[35] Wager E., Williams P., on behalf of the OPEN Project (Overcome failure to Publish nEgative fiNdings) Consortium (2013). “Hardly worth the effort”? Medical journals’ policies and their editors’ and publishers’ views on trial registration and publication bias: Quantitative and qualitative study. BMJ.

[36] Mathieu S., Chan A.W., Ravaud P. (2013). Use of trial register information during the peer review process. PLoS ONE.

[37] van Lent M., IntHout J., Out H.J. (2015). Differences between information in registries and articles did not influence publication acceptance. J. Clin. Epidemiol..

[38] Omar I.A., Jayaraman R., Salah K., Simsekler M.C.E., Yaqoob I., Ellahham S. (2020). Ensure protocol compliance and data transparency in clinical trials using Blockchain smart contract. BMC Med. Res. Methodol..

[39] Al-Durra M., Nolan R.P., Seto E., Cafazzo J.A. (2020). Prospective registration and reporting of trial number in randomised clinical trials: Global cross sectional study of the adoption of ICMJE and Declaration of Helsinki recommendations. BMJ.

[40] Knowles R.L., Ha K.P., Mueller J., Rawle F., Parker R. (2020). Challenges for funders in monitoring compliance with policies on clinical trials registration and reporting: Analysis of funding and registry data in the UK. BMJ Open.

[41] Jones C.W., Adams A.C., Murphy E., King R.P., Saracco B., Stesis K.R. (2021). Delays in reporting and publishing trial results during pandemics: Cross sectional analysis of 2009 H1N1, 2014 Ebola, and 2016 Zika clinical trials. BMC Med. Res. Methodol..

[42] Xu Z., Li M. (2019). Statistical considerations for bias and protocol deviation in medical device pivotal clinical study. Ther. Innov. Regul. Sci..

